# Mn-X (X = F, Cl, Br, I) Co-Doped GeSe Monolayers: Stabilities and Electronic, Spintronic and Optical Properties

**DOI:** 10.3390/nano13121862

**Published:** 2023-06-15

**Authors:** Wenjie He, Xi Zhang, Dan Gong, Ya Nie, Gang Xiang

**Affiliations:** College of Physics, Sichuan University, Chengdu 610065, China; 13980505233@163.com (W.H.); 15183568892@163.com (D.G.); nieya1104@scu.edu.cn (Y.N.)

**Keywords:** GeSe, co-doping, carrier mobility, magnetization, optical property

## Abstract

GeSe monolayer (ML) has recently attracted much interest due to its unique structure and excellent physical properties that can be effectively tuned through single doping of various elements. However, the co-doping effects on GeSe ML are rarely studied. In this study, the structures and physical properties of Mn-X (X = F, Cl, Br, I) co-doped GeSe MLs are investigated by using first-principle calculations. The formation energy and phonon disspersion analyses reveal the stability of Mn-Cl and Mn-Br co-doped GeSe MLs and instability of Mn-F and Mn-I co-doped GeSe MLs. The stable Mn-X (X = Cl, Br) co-doped GeSe MLs exhibit complex bonding structures with respect to Mn-doped GeSe ML. More importantly, Mn-Cl and Mn-Br co-doping can not only tune magnetic properties, but also change the electronic properties of GeSe MLs, which makes Mn-X co-doped GeSe MLs indirect band semiconductors with anisotropic large carrier mobility and asymmetric spin-dependent band structures. Furthermore, Mn-X (X = Cl, Br) co-doped GeSe MLs show weakened in-plane optical absorption and reflection in the visible band. Our results may be useful for electronic, spintronic and optical applications based on Mn-X co-doped GeSe MLs.

## 1. Introduction

Since the experimental realization of graphene [1], two-dimensional (2D) materials have attracted much interest and have been heavily investigated, such as black phosphorene (BP) [2], hexagonal boron nitride (h-BN) [3], group IV monolayers (MLs) [4], transition-metal dichalcogenide (TMD) MLs [4], metal dichalcogenide (MD) MLs [5,6], group IV-V MLs [7], group IV monochalcogenide MLs [8], group III-V MLs [9] and ternary Bi oxyhalide MLs [10]. Among them, with a similar structure to that of BP, group VI monochalcogenide (MX, M = Ge, Sn; X = S, Se) MLs have attracted increasing attention for their excellent optical and electrical properties [8,11,12,13,14]. In contrast to most MX MLs with indirect band gaps, GeSe ML is a p-type semiconductor with a direct band gap of 1.16 eV [12,15] and exhibits a carrier mobility as high as 6.22 × 10^3^ cm^2^v^−1^s^−1^ [11], which is desirable for electrical and optical devices. Furthermore, the physical properties of GeSe ML can be modulated effectively through single doping of diverse elements. For instance, GeSe ML can be turned from nonmagnetic to magnetic through single doping of transition-metal (TM) elements [16], among which Mn doping introduces the largest magnetic moment of 5.0 μB per dopant [17]. Meanwhile, single doping of halogen element X (X = F, Cl, Br, I) can significantly modify the electrical properties of GeSe ML by introducing additional carriers [18]. It is noted that Li et al. studied the co-doping effect of Zn-Ga on the thermoelectric properties of 2D group VI monochalcogenide SnSe nanosheets [19]. However, there are no reports on Mn-X co-doping effects on the electronic, magnetic and optical properties of 2D GeSe MLs, which may be useful for the design and application of GeSe-based materials and devices.

In this work, we have investigated the stabilities and structures and electronic, magnetic and optical properties of Mn-X (X = F, Cl, Br, I) co-doped GeSe MLs by using first-principle calculations. The phonon dispersion curves revealed the stability of Mn-Cl and Mn-Br co-doped GeSe MLs and the instability of Mn-F and Mn-I co-doped GeSe MLs. Further calculations showed that the magnetic, electronic and optical properties of GeSe MLs were tuned by the Mn-X co-doping simultaneously. Our results provide insight into the Mn-X co-doping effects on the GeSe MLs and may be useful for the design, fabrication and application of Mn-X co-doping group IV monochalcogenide MLs.

## 2. Computational Methods

The calculations were performed using the Vienna ab initio Simulation Package (VASP) based on density functional theory (DFT) [20]. Both the properties of undoped and doped GeSe MLs were optimized with the projected augmented wave (PAW) method [21]. The Perdew-Burke-Ernzerhof (PBE) function of the generalized gradient approximation (GGA) was used as the exchange correlation function [22]. The simulations were done in a 2 × 2 supercell GeSe ML with 8 Ge and 8 Se atoms, in which 1 Mn and 1 X atoms are doped, corresponding to a doping concentration of 6.25% for each element. We chose a 3 × 3 supercell in addition to a 2 × 2 supercell to perform the calculations to evaluate the influence of material size and doping concentration. We used the projector augmented-wave pseudopotential method with a plane-wave basis set with a kinetic-energy cutoff of 500 eV. The Monkhorst-Pack scheme was used in the Brillouin zone with k-point meshes of 11 × 11 × 1 throughout the calculations. The thickness of the vacuum region was kept greater than 15 Å. Structural relaxation was carried out until the total energy converged to 10^−6^ eV and the force on each atom was less than 0.01 eV/Å. Gaussian smearing was used with a small width of 0.01 eV. For describing the strong correlation interaction of d electrons, the GGA + U method was used with a U value of 3.0 eV for the Mn atom [16].

## 3. Results and Discussions

**Figure 1:**
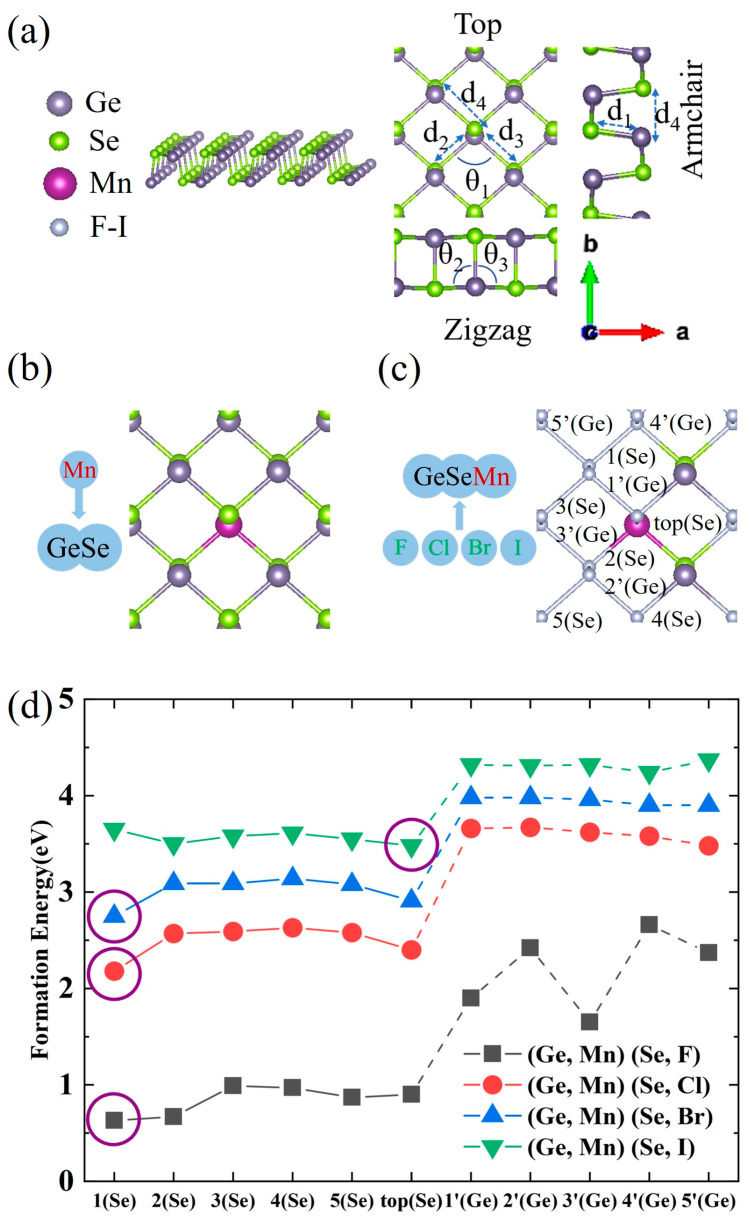
(**a**) The atomic structure of GeSe ML. (**b**) The doping site of Mn in Mn-doped GeSe ML. (**c**) The possible doping sites of X in Mn-X co-doped GeSe MLs. (**d**) The formation energies of Mn-X co-doped GeSe MLs as a function of doping sites. The doping sites with the lowest energy formation are circled in purple.

Figure 1a shows the structure of a 2 × 2 GeSe ML supercell. The lattice constants were calculated to be a = 3.99 Å and b = 4.26 Å in GeSe primitive cell, as shown in Table 1, consistent with previous theoretical results [12,23], although slightly different from experimental results [24], in which b (10.840 Å) is the lattice parameter along the layers, while a (4.394 Å) corresponds to our b (4.26 Å) and c (3.833 Å) corresponds to our a (3.99 Å). The Mn-X co-doping process was performed as follows. The Mn doping position was first optimized, and then the X doping position was considered and optimized, as shown in Figure 1b,c. The formation energy *E_form_* of the co-doped GeSe ML was calculated using the following formula: [25] *E_form_ = E_doped_* − *E_undoped_ + E_sites_* − *E_dopants_*, where *E_doped_* and *E_undoped_* are the total energies of the GeSe ML with and without dopant atoms, respectively, *E_sites_* is the sum of the energy of substituted Ge and Se atoms, and *E_dopants_* is the sum of the energy of the Mn and X dopants. It was found that the *E_form_* value (0.85 eV) of Mn-doped GeSe ML obtained from Mn substituting Ge is smaller than that (3.33 eV) from Mn substituting Se, meaning that Mn tends to occupy the Ge site, consistent with previous theoretical studies [16]. Figure 1d shows that, given that Mn is substituting Ge, the formation energies of X atoms doped at the Se sites are generally lower than those at the Ge sites in the Mn-X co-doping, indicating that X atoms tend to occupy the Se sites. Specifically, F, Cl and Br tend to occupy the 1(Se) site (Figure 1c) adjacent to the Mn sites, while I tends to occupy the top (Se) site (Figure 1c) opposite to the Mn site, probably due to its larger atomic mass. Based on the results above, for convenience, Mn-doped GeSe ML is named (Ge, Mn) Se ML, while Mn-X co-doped GeSe ML is named (Ge, Mn) (Se, X) ML.

**Figure 2:**
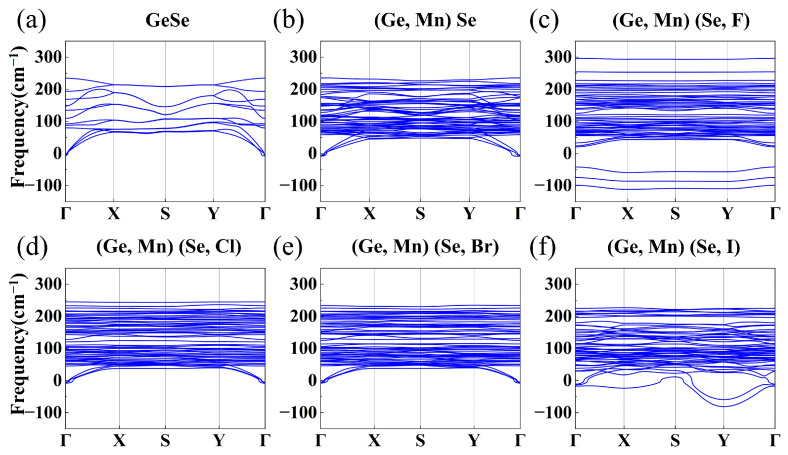
(**a**–**f**) The phonon dispersion curves of undoped, Mn-doped and Mn-X co-doped GeSe MLs.

To further explore the thermodynamic stability of undoped, Mn-doped and Mn-X co-doped GeSe MLs, the phonon dispersion curves were calculated by using VASP and PHONOPY packages based on the density functional perturbation theory (DFPT), ref. [26] as shown in Figure 2a–f, where the absence of imaginary frequencies indicates the stability of the materials. The phonon dispersion curves of GeSe ML show three acoustic branches and nine optical branches, which is consistent with previous theoretical calculations [27,28]. As shown in Figure 2, all undoped and doped GeSe MLs are stable, except for Mn-F and Mn-I co-doped GeSe MLs. Their instability may be attributed to the overly active outer electrons of the F atom and the large effective mass of the I atom. From the phonon spectra, it was observed that Mn-F and Mn-I co-doping rendered GeSe monolayers unstable. This observation may save experimenters time in related attempts and also remind them to choose doping elements more carefully in similar co-doping systems. Therefore, the relevant properties of Mn-F and Mn-I co-doped GeSe MLs will not be discussed. 

Subsequently, the doping effects on the structure of GeSe MLs were investigated. The calculated structural parameters of undoped and doped GeSe MLs are listed in Table 1. The changes in lattice constants *a* and *b* indicated that Mn-X co-doping generally had a larger impact on the structure than single doping of Mn, which is also shown in Figure 2b–f. In addition, (Ge, Mn) Se ML’s *d*_2_ and *d*_3_ were equal to GeSe ML’s *d*_2_ and *d*_3_, while Mn-X co-doped GeSe MLs showed obvious variations in *d*_2_ and *d*_3_, again suggesting that co-doping has a stronger effect on the structure of GeSe ML. *d*_4_, the bond length between Mn atom and X atom in (Ge, Mn) (Se, X) (X = Cl, Br) ML, was 2.61 Å and 2.79 Å for Mn-Cl and Mn-Br co-doping, respectively, revealing the influence of electronegativity of halogen elements on the structure of GeSe MLs. 

**Figure 3:**
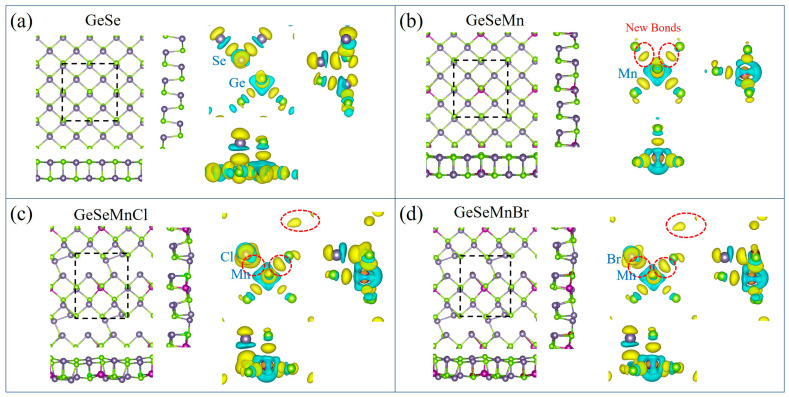
(**a**–**d**) The fully optimized structures and charge density differences of undoped, Mn-doped, Mn-Cl co-doped and Mn-Br co-doped GeSe MLs, respectively. The supercells are outlined using a black-dotted box. The new bands are outlined using red-dotted circles. Navy balls, green balls and purple balls denote Ge atoms, Se atoms and Mn atoms, respectively, while the other colors represent different X atoms. For charge density differences, the isosurface value is selected at 0.004 eV/Å. The yellow color and blue color denote electron the accumulation region and depletion region, respectively.

The bonding structures of undoped and doped GeSe MLs were revealed by charge density differences (Δρ = ρ_doped_ − ρ_vacancy_ − ρ_dopant_), as shown in Figure 3a–d. In single Mn doping, the Mn atom formed five bonds with the surrounding Ge atoms. In Mn-X co-doping, the Mn atom formed five bonds with an X atom and four Se atoms, while the X atom formed two bonds with the Mn and Ge atoms. When the X atom replaced a Se atom, the Ge-Se bonds were broken, and the Mn-X bond was formed, which further rearranged the charge density and affected the structure. The structure of the Mn-X co-doped GeSe monolayer was more variable than that of Mn single doping. The position of the X atom changed to a certain extent, and a hole formed around it due to the lack of bonds, which resulted in more peculiar properties due to structural changes. In addition, some new bonds were formed far away from the Mn and X (Cl, Br) atoms, which may explain (Ge, Mn) (Se, X) ML’s strange electrical and magnetic properties.

**Figure 4:**
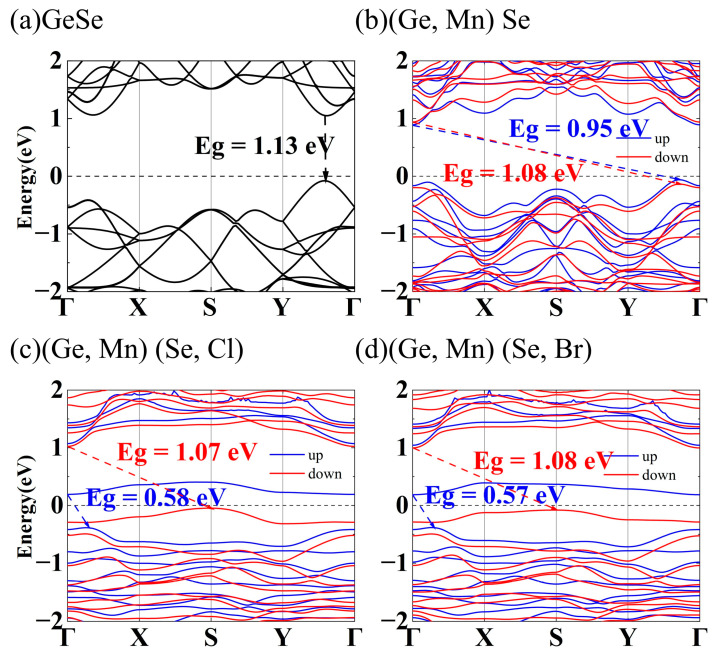
The band structures of (**a**) undoped, (**b**) Mn-doped, (**c**) Mn-Cl co-doped and (**d**) Mn-Br co-doped GeSe MLs.

The electronic band structures of the undoped, Mn-doped and Mn-X (Cl, Br) co-doped GeSe MLs were then studied. Figure 4a shows that GeSe ML possesses a direct band gap of 1.13 eV, where the valence-band maximum (VBM) and the conduction-band minimum (CBM) lie along the Γ-Y direction, consistent with previous theoretical results (1.16 eV) [12,15]. The spin dependent band structures in Figure 4b–d show that (Ge, Mn) Se, (Ge, Mn) (Se, Cl) and (Ge, Mn) (Se, Br) MLs are indirect semiconductors. As shown in Figure 4 and Table 2, the up-spin bands of (Ge, Mn) Se, (Ge, Mn) (Se, Cl) and (Ge, Mn) (Se, Br) MLs exhibit band gap values of 0.95 eV, 0.58 eV and 0.57 eV, respectively, smaller than the band gap value of GeSe ML, while both (Ge, Mn) (Se, Cl) and (Ge, Mn) (Se, Br) MLs are tuned to be n-type semiconductors. The down-spin bands of (Ge, Mn) Se, (Ge, Mn) (Se, Cl) and (Ge, Mn) (Se, Br) MLs exhibit band gaps of 1.08 eV, 1.07 eV and 1.08 eV, respectively. The asymmetry between the up-spin and down-spin band structures of (Ge, Mn) (Se, Cl) and (Ge, Mn) (Se, Br) MLs demonstrates their potential in semiconductor spintronic applications. 

To investigate the effect of different U values on the electronic structure of our systems, the bands of Mn-doped, Mn-Cl and Mn-Br co-doped GeSe MLs with the previously used U values of 4.0 eV, 5.0 eV and 6.0 eV [29,30,31], in addition to the U value of 3.0 eV, were calculated and shown in Figure S1. The band structures were nearly consistent, indicating that our systems were insensitive to the U values above 3.0 eV. For a clearer comparison, the bandgap values and their relative change ratios are listed in Table S1, where the maximum change ratio is as low as 2.88%, indicating that a U value of 3.0 eV is sufficient for the Mn atom in our systems.

**Figure 5:**
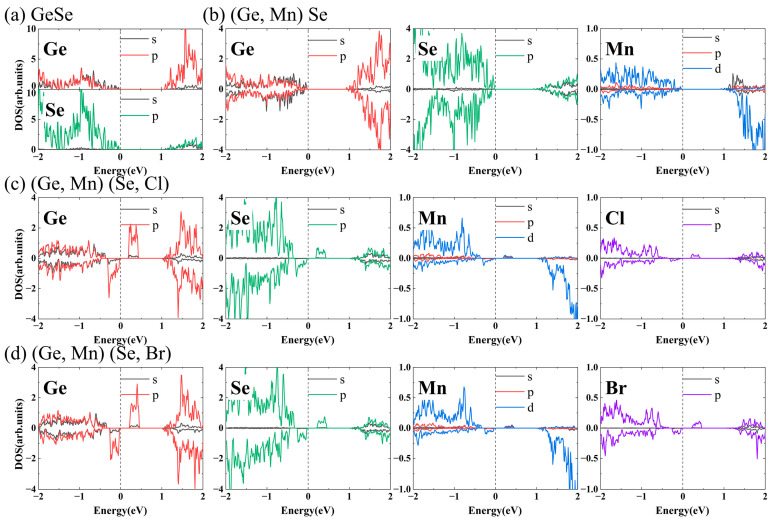
PDOSs of (**a**) undoped, (**b**) Mn-doped, (**c**) Mn-Cl co-doped and (**d**) Mn-Br co-doped GeSe MLs.

In order to clarify contributions from different orbitals to band structures around the Fermi level, the partial density of states (PDOSs) of undoped and doped GeSe MLs are shown in Figure 5. In GeSe ML, CBM and VBM were contributed by Ge p and Se p orbital, respectively. In (Ge, Mn) Se ML, CBM and VBM of the up-spin bands were contributed by Mn s and d orbitals, respectively, while CBM and VBM of the down-spin bands were contributed by Mn d orbital. Ge and Se p orbitals hybridized with the Mn d orbital and contributed significantly to the CBM and VBM. In (Ge, Mn) (Se, Cl) and (Ge, Mn) (Se, Br) MLs, impurity levels were contributed by Cl p and Br p orbitals, and were close to the CBMs in the up-spin bands and close to the VBMs in the down-spin bands, respectively. Ge and Se p orbitals also played an important role in impurity levels due to hybridization with Ge, Se, X (Cl, Br) p orbitals and Mn d orbital.

Since undoped, Mn-doped, Mn-Cl co-doped and Mn-Br co-doped GeSe MLs are semiconductors and carrier mobility is one of the key parameters of semiconductors [32], the electron and hole carrier mobility values of them were calculated using the deformation potential theory two dimensional (2D) semiconductors [33,34], as follows:

$$\mu_{2D}=\frac{2e\hbar^{3}C_{2D}}{3k_{B}Tm_{e}^{*}m_{d}E_{l}^{2}}$$

where $C_{2D}$ is elastic modulus of the strain, derived from $C_{2D}=\frac{\left(\frac{\partial^{2}E}{\partial^{2}\left(\frac{\Delta l}{l_{0}}\right)}\right)}{S}$, in which E is total energy of systems; Δl is a small change in the lattice constant $l_{0}$; S is the area of lattice for a 2D system; T is temperature (300 K); $m_{e}^{*}\;$is the effective mass of the carrier along the transport direction; and $m_{d}\;$is the average effective mass, derived from $m_{d}=\sqrt{\left(m_{a}^{*}m_{b}^{*}\right)}$; and $E_{l}$ is the deformation potential, derived from $E_{l}=\frac{\Delta E}{\left(\frac{\Delta l}{l_{0}}\right)}$, in which ΔE is energy change of CBM or VBM under strain. As shown in Table 3, the carrier mobility of GeSe ML in the b direction reaches 7494 cm^2^v^−1^s^−1^, similar to previous theoretical results [11,12]. The maximum carrier mobility values of (Ge, Mn) Se, (Ge, Mn) (Se, Cl) and (Ge, Mn) (Se, Br) MLs were 5603 cm^2^v^−1^s^−1^, 9060 cm^2^v^−1^s^−1^, and 3652 cm^2^v^−1^s^−1^, respectively, indicating that the MLs can keep high carrier mobility even after Mn-Cl and Mn-Br co-doping. The main carriers (holes) mobility values of (Ge, Mn) (Se, Cl) and (Ge, Mn) (Se, Br) MLs in a spin-down state were 107 cm^2^v^−1^s^−1^, 14 cm^2^v^−1^s^−1^ in the a direction and 16 cm^2^v^−1^s^−1^, 2 cm^2^v^−1^s^−1^ in the b direction, respectively, The main carrier (electrons) mobility values of (Ge, Mn) (Se, Cl) and (Ge, Mn) (Se, Br) MLs in a spin-up state were 673 cm^2^v^−1^s^−1^, 2987 cm^2^v^−1^s^−1^ in the a direction and and 8 cm^2^v^−1^s^−1^, 20 cm^2^v^−1^s^−1^ in the b direction, respectively, indicating the anisotropic nature of carrier mobility following Mn-Cl and Mn-Br co-doping. Interestingly, following Mn-Br co-doping, GeSe ML’s main carriers in a spin-up state changed from holes (450 cm^2^v^−1^s^−1^) to electrons (2987 cm^2^v^−1^s^−1^). 

**Figure 6:**
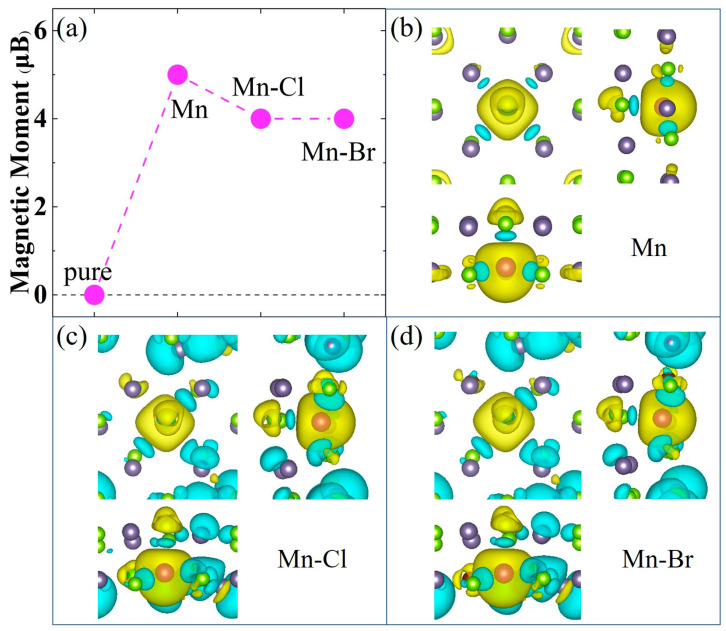
(**a**) Magnetic moments as the function of the undoped and doped GeSe MLs. Spin charge density of (**b**) Mn-doped, (**c**) Mn-Cl co-doped and (**d**) Mn-Br co-doped GeSe MLs. The yellow and blue represent the spin-up and spin-down electrons distribution, and the isosurface value is set at 0.001 eV/Å.

The magnetic properties of the Mn-doped and Mn-X co-doped GeSe MLs were then investigated. The calculated magnetic moments and spin charge density (Δρ = ρ_up_ − ρ_down_) distributions in Figure 6 show that all the doped GeSe MLs are magnetic. Our results indicate that single doping of Mn results in a magnetic moment of 5.0 μB in (Ge, Mn) Se ML, which mainly comes from the spin-up electrons around Mn, consistent with previous theoretical results [16,17] and slightly larger than the 4.25 μB observed in GeMnSe nanocombs in previous experimental studies [35]. Mn-Cl and Mn-Br co-doping further weakened the magnetic moment to 4.0 μB in both cases by introducing more spin-down electrons in (Ge, Mn) (Se, Cl) and (Ge, Mn) (Se, Br) MLs. The spin-down electrons that produce changes at sites far away from the Mn and X (Cl, Br) atoms significantly correlated with new bonds, as shown in Figure 3.

**Figure 7:**
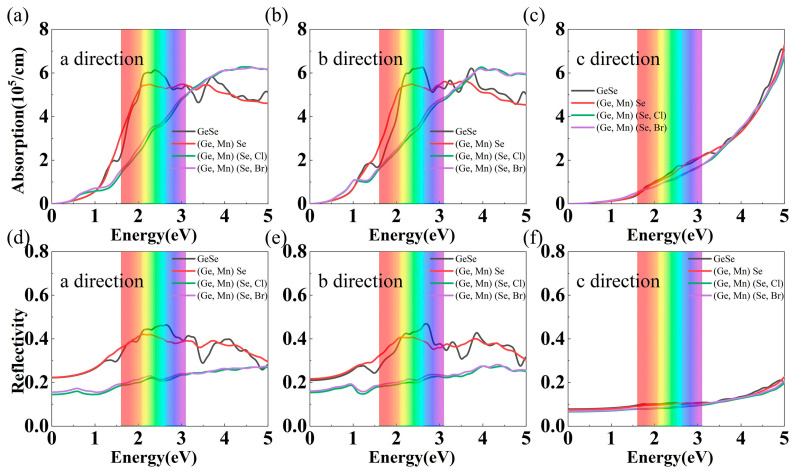
Light absorption of undoped, Mn-doped and Mn-X co-doped GeSe MLs along (**a**) the a direction, (**b**) the b direction and (**c**) the c direction. Light reflectivity of undoped, Mn-doped and Mn-X co-doped GeSe MLs along (**d**) the a direction, (**e**) the b direction and (**f**) the c direction.

The optical properties of undoped and doped GeSe MLs were then investigated. The optical absorption and reflectivity of undoped and doped GeSe MLs were almost the same in the a and b directions (the in-plane directions), as shown in Figure 7. The optical properties of Mn-doped GeSe ML were similar to those of GeSe ML. With respect to those of undoped GeSe ML, the in-plane optical absorption and reflection of Mn-Cl and Mn-Br co-doped GeSe MLs in the visible band were weakened but remained at the same order of magnitude. The optical absorption and reflection of Mn-Br co-doped GeSe ML were slightly stronger than those of Mn-Cl co-doped GeSe ML. Weaker optical absorption and reflection in the visible band suggest that Mn-X co-doped GeSe MLs could be useful as transparent materials. In the ultraviolet band, the in-plane optical absorption of Mn-Cl and Mn-Br co-doped GeSe MLs was strengthened, while the in-plane optical reflection remained weak. Stronger optical absorption and weaker reflection in the ultraviolet band suggested that Mn-X co-doped GeSe MLs could be useful for ultraviolet light absorption.

Finally, to evaluate the influence of material size and doping concentration, we chose a 3 × 3 supercell in addition to a 2 × 2 supercell to perform the calculations. We calculated undoped, Mn-doped, and Mn-X co-doped GeSe monolayers in 3 × 3 supercells, which corresponded to a doping concentration of 2.78% for each element. We did not change the parameters of the calculation, except for using the Monkhorst-Pack scheme in the Brillouin zone with k-point meshes of 7 × 7 × 1 throughout all the calculations in 3 × 3 supercells, to ensure consistent accuracy. Then the band structures of the undoped and doped GeSe monolayers under the 2 × 2 and 3 × 3 supercells were compared, as shown in Figure S2. For GeSe ML in 3 × 3 supercells, compared to that in 2 × 2 supercells, the bandgap type remained direct and the bandgap changed slightly from 1.13 eV to 1.14 eV. For doped GeSe monolayers, the bandgap type remained indirect. However, for Mn-doped, Mn-Cl co-doped and Mn-Br co-doped GeSe Monolayers, the bandgap changed by 0.15 eV, 0.08 eV and 0.09 eV in the spin-up states, and by 0.12 eV, 0.09 eV and 0.12 eV in spin-down states, respectively. After enlarging the supercells, the variations of the band gaps ranged from 8.4% to 15.8%, indicating that the doping concentration had a small effect on the bandgap, as shown in Table S2. To examine the contributions of different orbitals to the band structures, the PDOS of undoped and doped GeSe Monolayers were studied using supercells of two sizes, as shown in Figure S3. We found that since the numbers of Ge and Se atoms were almost doubled, the contributions of their orbitals to the bands were also nearly doubled. From the PDOS, our conclusions about which elements and which orbitals contributed to the VBM and CBM remain unchanged. It is worth noting that the contributions of the Ge p and Se p orbitals to the impurity levels were not doubled, indicating that the asymmetric spin-dependent band structures were mainly derived from the Mn and X atoms and their nearby Ge and Se atoms. The change in doping concentration did not change this characteristic feature. The spin charge densities of undoped and doped GeSe Monolayers are shown in Figure S4. In 3 × 3 supercells, the magnetic moment of Mn-doped GeSe ML mainly came from the spin-up electrons around Mn. Mn-Cl and Mn-Br co-doping introduced more spin-down electrons at sites far away from the Mn and X (Cl, Br) atoms. In different supercells, our conclusions regarding the origin of the magnetic properties in doped GeSe monolayers remain unchanged. As shown in Table S3, the magnetic moments remain consistent in different sized supercells, which are 5 μB, 4 μB and 4 μB for Mn doped, Mn-Cl co-doped and Mn-Br co-doped GeSe monolayers, respectively. Finally, we investigated the light absorption and reflection of undoped and doped GeSe monolayers in different-sized supercells, as shown in Figure S5. In 3 × 3 supercells, the reduced light absorption and reflection capacity in the visible band of Mn-X co-doped GeSe monolayers were restored, meaning that the optical properties of Mn-X co-doped GeSe monolayers were sensitive to doping concentrations. Moreover, the differences in the optical properties of Mn-Cl and Mn-Br co-doped GeSe monolayers became more pronounced, indicating that optical properties with different doping elements had different sensitivity to doping concentrations. By tuning different doping concentrations, different optical properties can be achieved, showing potential in optoelectronic device applications. 

## 4. Conclusions

In summary, we have systematically investigated the stabilities, structures and electrical, transport, magnetic and optical properties of transition metal Mn and halogen elements (X = F, Cl, Br, I) co-doped GeSe MLs by using first-principle calculations. Our results reveal the instability of Mn-X (X = F, I) co-doped GeSe MLs and the stability of Mn-X (X = Cl, Br) co-doped GeSe MLs. Further calculations show that Mn-X (X = Cl, Br) co-doped GeSe monolayers are indirect band semiconductors with anisotropic large carrier mobility and asymmetric spin-dependent band structures. Compared to Mn doping, co-doping with Mn-Cl and Mn-Br can not only adjust the magnetic moments (from 5 μB to 4 μB) but also alter the electronic properties of GeSe monolayers. Furthermore, Mn-X (X = Cl, Br) co-doped GeSe monolayers exhibit weakened in-plane optical absorption and reflection in the visible band, which may be useful for optical applications. Our results give insights into the Mn-X co-doping effects on the structures and physical properties of GeSe MLs and may be useful for related electronic, spintronic and optical applications.

## Figures and Tables

**Table 1:** The lattice constants (*a* and *b*), bond angles (*θ*_1_, *θ*_2_ and *θ*_3_) and bond lengths (*d*_1_, *d*_2_, *d*_3_ and *d*_4_) of undoped and doped GeSe MLs.

Dopant	*a* (Å)	*b* (Å)	*θ*_1_ *˚*	*θ*_2_ *˚*	*θ*_3_ *˚*	*d*_1_ (Å)	*d*_2_ (Å)	*d*_3_ (Å)	*d*_4_ (Å)
None	7.97	8.52	96.38	97.53	97.53	2.55	2.68	2.68	
Mn	7.96	8.27	92.96	101.55	101.55	2.57	2.68	2.68	2.86
Mn-Cl	7.84	9.19	89.60	108.49	97.18	2.59	2.62	2.79	2.61
Mn-Br	7.87	9.17	90.42	107.85	97.70	2.59	2.61	2.78	2.79

**Table 2:** The band gaps of undoped and doped GeSe MLs, where D and I demonstrate direct and indirect band gaps, respectively.

Dopant	Spin	Bandgap (eV)
None	up	1.13(D)
	down	1.13(D)
Mn	up	0.95(I)
	down	1.08(I)
Mn-Cl	up	0.58(I)
	down	1.07(I)
Mn-Br	up	0.57(I)
	down	1.08(I)

**Table 3:** Effective mass m (with m_0_ being the static electron mass), deformation potential constant E, elastic modulus C, and carrier mobility μ along the a and b directions. The electron and hole carrier mobility μ values are calculated at T = 300 K.

Dopant	Spin	Carrier	m_a_ (m_0_)	m_b_ (m_0_)	C_a_ (N/m)	C_b_ (N/m)	E_a_ (eV)	E_b_ (eV)	μ_a_ (cm^2^v^−1^s^−1^)	μ_b_ (cm^2^v^−1^s^−1^)
None		electron	0.37	0.13	45.56	22.65	6.77	1.22	172	7494
		hole	0.31	0.14	45.56	22.65	10.18	4.92	96	450
Mn	up	electron	0.14	0.18	49.84	35.13	4.26	2.02	1735	4230
		hole	0.32	0.18	49.84	35.13	7.06	5.80	183	339
	down	electron	0.16	0.19	49.84	35.13	2.59	1.63	3739	5603
		hole	0.30	0.24	49.84	35.13	7.66	4.76	148	338
Mn-Cl	up	electron	1.79	6.94	29.05	33.79	0.31	1.52	673	8
		hole	0.42	0.63	29.05	33.79	4.12	3.35	111	130
	down	electron	0.24	0.4	29.05	33.79	3.23	0.65	526	9060
		hole	1.39	2.27	29.05	33.79	1.24	2.69	107	16
Mn-Br	up	electron	1.57	1.88	30.36	17.58	0.23	1.96	2987	20
		hole	0.42	0.62	30.36	17.58	4.51	3.44	98	66
	down	electron	0.26	0.35	30.36	17.58	2.4	0.8	944	3652
		hole	2.58	6.39	30.36	17.58	1.68	2.42	14	2

## Data Availability

The data supporting the findings of this study are available from the authors upon reasonable and appropriate request.

## Supplementary Materials

The following supporting information can be downloaded at: https://www.mdpi.com/article/10.3390/nano13121862/s1. Figure S1: The band structures of (a) Mn doped, (b) Mn-Cl co-doped, and (c) Mn-Br co-doped GeSe Monolayers with different U values; Figure S2: In 2 × 2 and 3 × 3 supercell, the band structures of (a) undoped, (b) Mn-doped, (c) Mn-Cl co-doped and (d) Mn-Br co-doped GeSe Monolayers; Figure S3: In 2 × 2 and 3 × 3 supercell, PDOS of (a) undoped, (b) Mn-doped, (c) Mn-Cl co-doped and (d) Mn-Br co-doped GeSe Monolayers; Figure S4: Spin charge density of (a) Mn-doped, (b) Mn-Cl co-doped, (c) Mn-Br co-doped GeSe Monolayers in 2 × 2 supercell. Spin charge density of (d) Mn-doped, (e) Mn-Cl co-doped and (f) Mn-Br co-doped GeSe Monolayers in 3 × 3 supercell. The yellow and blue represent the spin-up and spin-down electrons distribution, and the isosurface value is set at 0.001 eV/Å; Figure S5: In 2 × 2 and 3 × 3 supercell, light absorption of undoped, Mn-doped and Mn-X co-doped GeSe Monolayers along (a) the a direction, (b) the b direction and (c) the c direction. Light reflectivity of undoped, Mn-doped and Mn-X co-doped GeSe Monolayers along (d) the a direction, (e) the b direction and (f) the c direction; Table S1: The band gaps of Mn doped, Mn-Cl, and Mn-Br co-doped GeSe Monolayers with different U values. I represents indirect band gap; Table S2: In 2 × 2 and 3 × 3 supercell, the band gaps of undoped and doped GeSe Monolayers, where D and I show direct and indirect band gaps; Table S3: The magnetic moments of doped GeSe Monolayers.
