# Supplementary material for: Mn-X (X = F, Cl, Br, I) Co-Doped GeSe Monolayers: Stabilities and Electronic, Spintronic and Optical Properties
Source: Nanomaterials (Basel). 2023 Jun 15;13(12):1862. doi: 10.3390/nano13121862 (PMC10301037; doi:10.3390/nano13121862)
Supplement: Supplementary file 1 [file nanomaterials-13-01862-s001.zip › nanomaterials-2419855-supplementary.pdf]

Supporting Information

Mn-X (X= F, Cl, Br, I) Co-doped GeSe Monolayers: Stabilities and Electronic, Spintronic and Optical Properties[1]

Wenjie He, Xi Zhang*, Dan Gong, Ya Nie, Gang Xiang*

College of Physics, Sichuan University, Chengdu 610065, China

***Corresponding authors' emails**: xizhang@scu.edu.cn; gxiang@scu.edu.cn

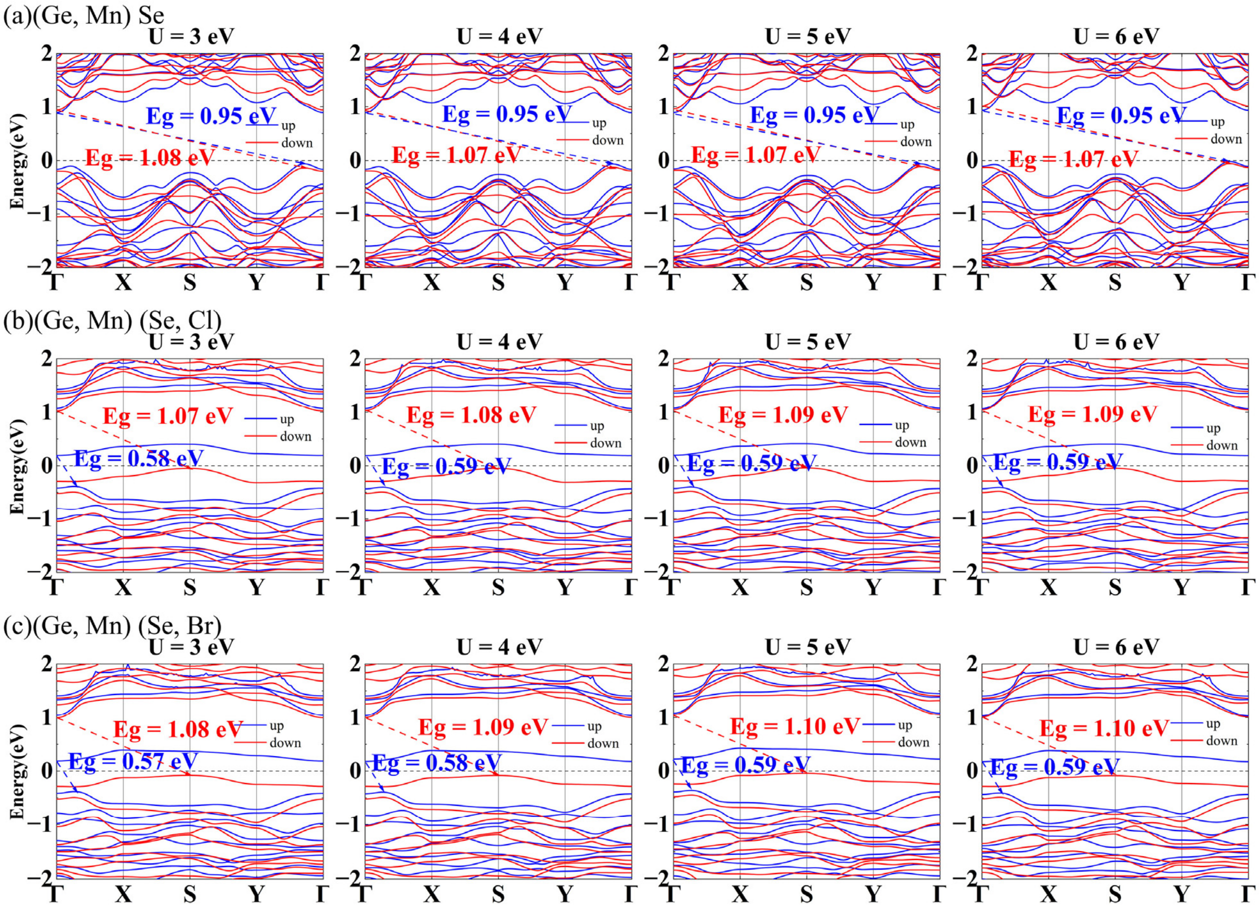

Figure. S1. The band structures of (a) Mn doped, (b) Mn-Cl co-doped, and (c) Mn-Br co-doped GeSe Monolayers with different U values.

[1] Project supported by the National Natural Science Foundation of China (Grant No. 52172272).

Table. S1. The band gaps of Mn doped, Mn-Cl, and Mn-Br co-doped GeSe Monolayers with different U values. I represents indirect band gap.

Dopant	U value (eV)	Spin	Bandgap (eV)	Change
Mn	3.0	up	0.9526(I)	0%
		down	1.0752(I)	0%
	4.0	up	0.9542(I)	0.17%
		down	1.0735(I)	-0.16%
	5.0	up	0.9546(I)	0.21%
		down	1.0723(I)	-0.27%
	6.0	up	0.9513(I)	-0.14%
		down	1.0675(I)	-0.72%
Mn-Cl	3.0	up	0.5779(I)	0%
		down	1.0709(I)	0%
	4.0	up	0.5890(I)	1.92%
		down	1.0797(I)	0.82%
	5.0	up	0.5918(I)	2.41%
		down	1.0864(I)	1.45%
	6.0	up	0.5930(I)	2.61%
		down	1.0913(I)	1.91%
Mn-Br	3.0	up	0.5694(I)	0%
		down	1.0771(I)	0%
	4.0	up	0.5750(I)	0.98%
		down	1.0856(I)	0.79%
	5.0	up	0.5851(I)	2.76%
		down	1.0961(I)	1.76%
	6.0	up	0.5858(I)	2.88%
		down	1.0987(I)	2.01%

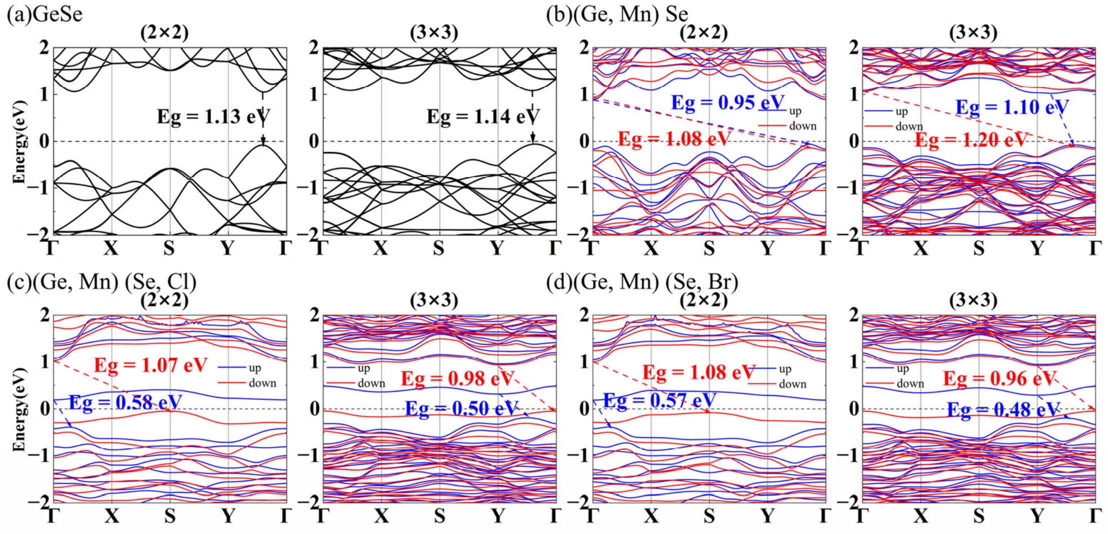

Figure S2. In 2×2 and 3×3 supercell, the band structures of (a) undoped, (b) Mn-doped, (c) Mn-Cl co-doped and (d) Mn-Br co-doped GeSe Monolayers.

Table S2. In 2×2 and 3×3 supercell, the band gaps of undoped and doped GeSe Monolayers, where D and I show direct and indirect band gaps.

Dopant	Spin	Bandgap(eV) (2×2)	Bandgap(eV) (3×3)	Change
None	up	1.13(D)	1.14(D)	0.9%
	down	1.13(D)	1.14(D)	0.9%
Mn	up	0.95(I)	1.10(I)	15.8%
	down	1.08(I)	1.20(I)	11.1%
Mn-Cl	up	0.58(I)	0.50(I)	13.8%
	down	1.07(I)	0.98(I)	8.4%
Mn-Br	up	0.57(I)	0.48(I)	15.8%
	down	1.08(I)	0.96(I)	11.1%

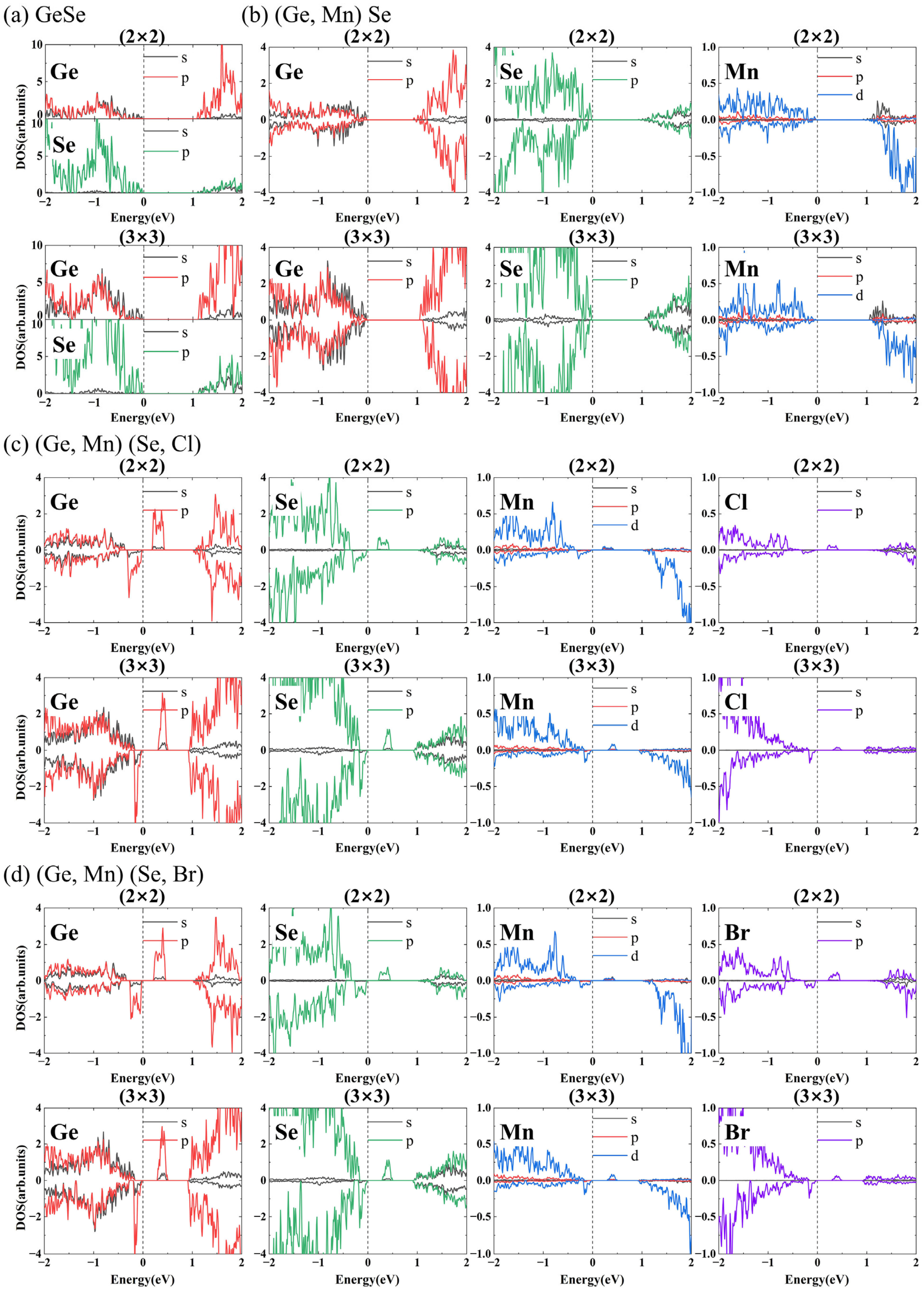

Figure S3. In 2×2 and 3×3 supercell, PDOS of (a) undoped, (b) Mn-doped, (c) Mn-Cl co-doped and (d) Mn-Br co-doped GeSe Monolayers.

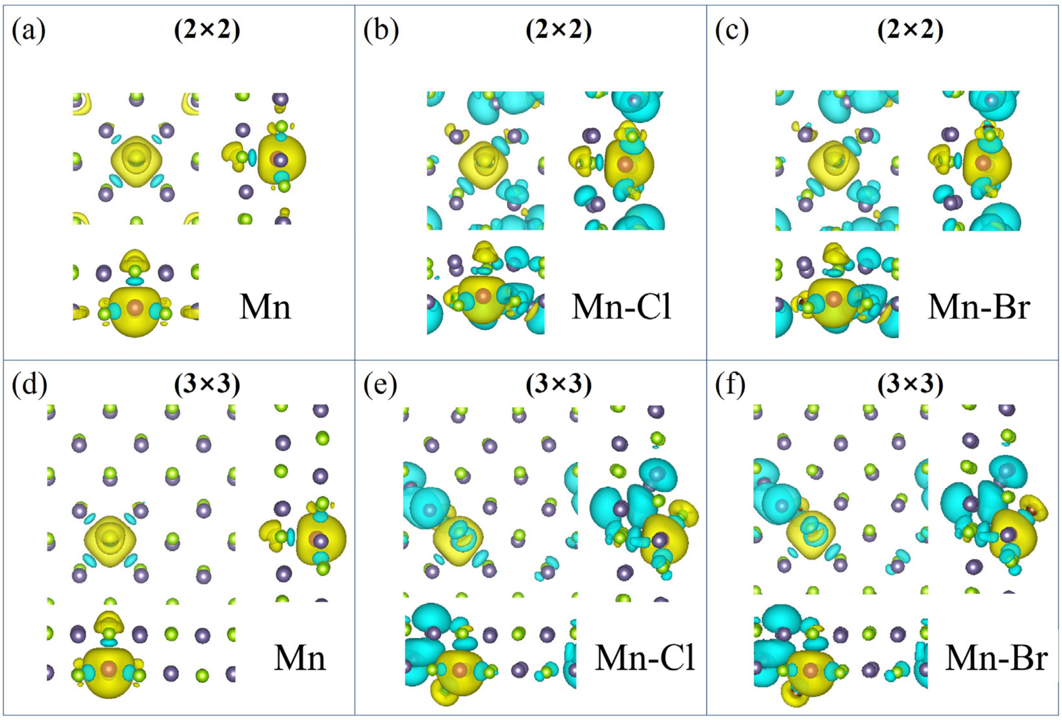

Figure S4. Spin charge density of (a) Mn-doped, (b) Mn-Cl co-doped, (c) Mn-Br co-doped GeSe Monolayers in 2×2 supercell. Spin charge density of (d) Mn-doped, (e) Mn-Cl co-doped and (f) Mn-Br co-doped GeSe Monolayers in 3×3 supercell. The yellow and blue represent the spin-up and spin-down electrons distribution, and the isosurface value is set at 0.001 eV/Å.

Table S3. The magnetic moments of doped GeSe Monolayers.

Dopant	Supercell	Magnetic Moments(μB)
Mn	2×2	5
	3×3	5
Mn-Cl	2×2	4
	3×3	4
Mn-Br	2×2	4
	3×3	4

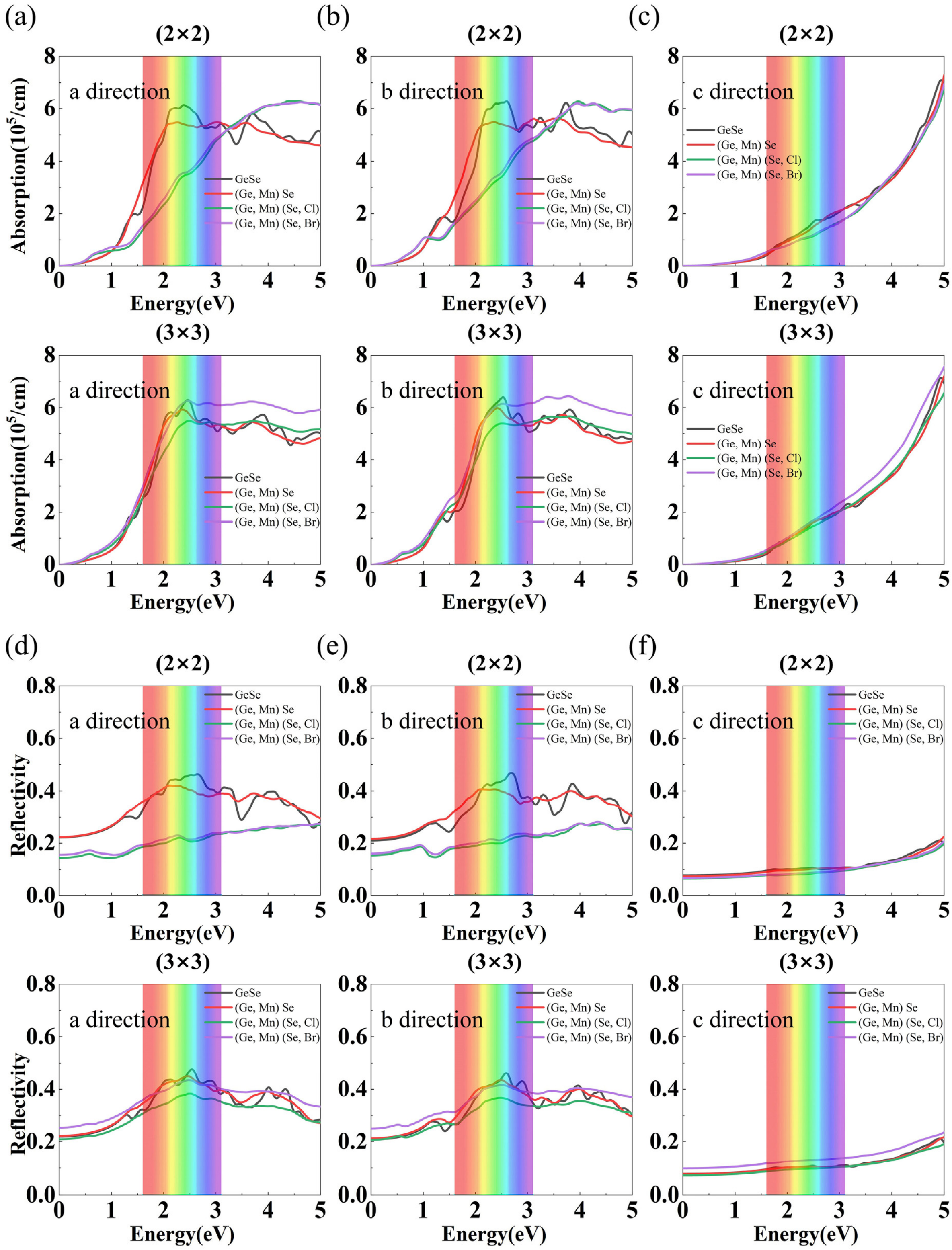

Figure S5. In 2×2 and 3×3 supercell, light absorption of undoped, Mn-doped and Mn-X co-doped GeSe Monolayers along (a) the a direction, (b) the b direction and (c) the c direction. Light reflectivity of undoped, Mn-doped and Mn-X co-doped GeSe Monolayers along (d) the a direction, (e) the b direction and (f) the c direction.
